# Distortions, deviations and alternative facts: reliability in crystallography

**DOI:** 10.1107/S205225252001458X

**Published:** 2021-01-01

**Authors:** William Clegg

**Affiliations:** a School of Natural and Environmental Sciences, Newcastle University, Newcastle upon Tyne NE1 7RU, United Kingdom

**Keywords:** structural distortions, structure interpretation, structure validation, scientific fraud

## Abstract

This feature article is derived from the author’s presentation of the Lonsdale lecture at the BCA Spring Meeting in 2018. One of the research results for which Kathleen Lonsdale is best known was her 1929 demonstration that the benzene ring in crystalline hexa­methyl­benzene is planar and has essentially hexagonal symmetry, resolving decades of dispute among organic chemists. More recent crystallographic studies of hexa­methyl­benzene have shown that there are actually small deviations from planarity. Such deviations for aromatic compounds may be due to electronic, steric, and/or intermolecular factors. Some substituted benzene molecules display remarkably large deviations, both from a planar ring structure and from regular hexagonal angular geometry around the ring. Starting from this specific connection with Kathleen Lonsdale’s research, a number of stories are recounted of structural distortions and deviations from expected results and explanations that have been suggested for them, across a wide range of chemical topics including macrocycles, metal clusters, unusual coordination geometry and isomerism. On the way we find genuine surprises and results that have led to new understanding, but also examples of poor experiments, misinterpretation of data, scientific bias and preconceived ideas, incompetence and even deliberate fraud. Some aspects of structure validation are discussed. While showcasing some interesting research in its own right, this account also serves an educational purpose.

## Abstract 2

In this text based on the 2018 Lonsdale lecture, beginning with early work by Kathleen Lonsdale, instructive examples are given of unusual and unexpected structures derived from X-ray crystallography, not all of which are genuine results.

## 1. Introduction   

**Figure 1:**
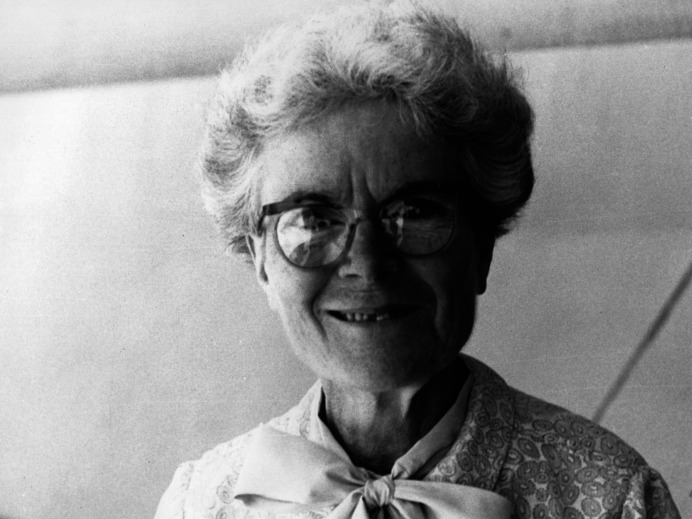
Professor Dame Kathleen Lonsdale (photographed in 1969; IUCr archive photograph number 24692).

This article is based on the Lonsdale lecture given by invitation at the British Crystallographic Association Spring Meeting at Warwick University in March 2018; by tradition, the Lonsdale Lecturer, nominated by the BCA Young Crystallographers’ Group, is expected to combine aspects of original research with an educational approach. The lecture in 2018, with a title partly inspired by a current political catchphrase, took its starting point from work carried out by Professor Dame Kathleen Lonsdale (Fig. 1 ▸), in whose honour the annual lecture was created.

Lonsdale, born Kathleen Yardley in 1903, was the first woman President of the IUCr (1966) and one of the first two women to be elected Fellow of the Royal Society (1945); she was appointed DBE (Dame Commander of the Most Excellent Order of the British Empire) in 1956. She died of cancer in 1971. Probably her best-known published work was as joint editor of Volume I (*Symmetry Groups*) of *International Tables for X-Ray Crystallography* in 1952. In addition to scientific works, she wrote books expressing her Christian faith and pacifism (as a Quaker), including an account of her time in prison as a conscientious objector, and based on her own balance of scientific research and family life; she was a full-time mother of three children in the early 1930s. She saw no conflicts in these diverse aspects of her life.

Lonsdale was concerned for both clarity and ethics in scientific research and training; in an article in *Nature* (Lonsdale, 1962 ▸) she expressed these concerns and prized the qualities of honesty, openness and humility in true scientists. These are themes reflected in this article.

Among Lonsdale’s early crystallographic research, she demonstrated that the ring of hexa­methyl­benzene is planar with essentially hexagonal symmetry (Lonsdale, 1928 ▸; 1929*a* ▸,*b* ▸). The structure was later improved by Brockway & Robertson (1939 ▸), but her original results were thereby simply confirmed with greater precision and more reliable bond lengths.

Is the ring *completely* planar? More recent and more precise investigations indicate that it is very nearly so, with small torsional twists to accommodate the steric congestion of six methyl groups; the ring itself is essentially planar within experimental uncertainties, while the substituents are displaced slightly above and below this plane. The Cambridge Structural Database (CSD; version 5.41, November 2019 with three updates to August 2020; Groom *et al.*, 2016 ▸) contains 23 entries for hexa­methyl­benzene itself (including deuterated structures) and 82 others in which it is a component, excluding metal-containing structures. For example, the entry HMBENZ04 (Le Magueres *et al.*, 2001 ▸) has a maximum internal torsion angle (C–C–C–C involving only ring atoms) of 2° and a maximum external torsion angle (Me–C–C–Me) of 5°, rounding to integer values.

## 2. Angular distortions in and around benzene rings   

Such out-of-plane distortions can be considerable with six bulky substituents. The most extreme case in which the substituents are chemically identical is C_6_(SiMe_3_)_6_ (KELVOM; Sakurai *et al.*, 1990 ▸) with internal torsion angles up to 12° and external up to 62°. A particularly distorted ring, with maximum internal and external torsions of 45 and 73°, respectively, is found in 1,3,5-tris­(di­ethyl­amino)-2,4,6-tri­nitro­benzene (JARLOD; Chance *et al.*, 1989 ▸); this is most certainly not a planar arrangement!

**Figure 2:**
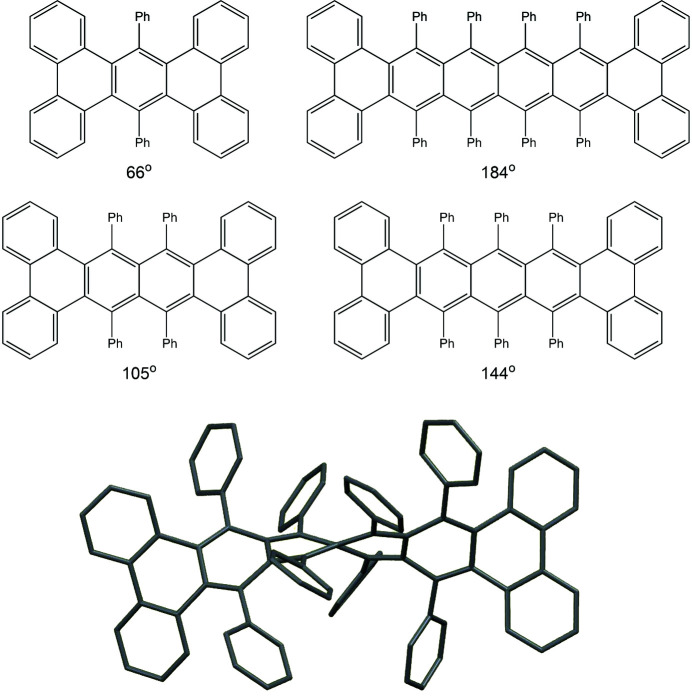
Twistacenes: twisted ribbons of fused benzene rings.

Polyaromatics, in which benzene rings are fused together, extend the scope for out-of-plane twists generated by steric hindrance of substituents on adjacent rings. This is well illustrated by a series of so-called ‘twistacenes’ (Fig. 2 ▸), in which the overall molecular twist is measured by the dihedral angle between the end groups; with just one di­phenyl-substituted ring this is already 66°, and increases with the addition of each further ring to 105, 144 and 184° (Pascal *et al.*, 1986 ▸; Rodríguez-Lojo *et al.*, 2013 ▸; Lu *et al.*, 2004 ▸; Clevenger *et al.*, 2018 ▸).

**Figure 3:**
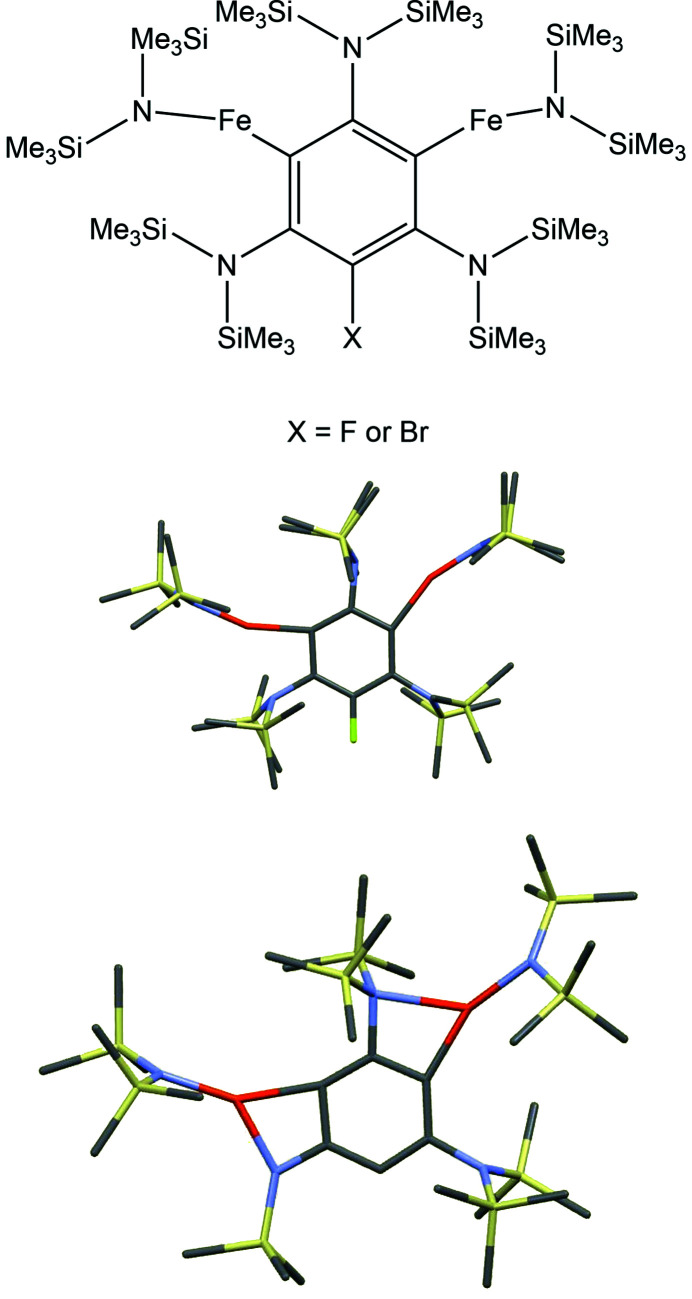
Ferrated benzene derivatives with extreme in-plane angular distortions.

Angular distortions may also occur in the plane of the ring; although ring internal angles generally deviate only slightly from the ideal value of 120°, the two external angles *X*—C—C for a substituent *X* can vary much more, one of them expanding while the other shrinks. Having found some marked distortions of this kind in our own research (Fig. 3 ▸, top and middle), I needed to find suitable structures for comparison and assessment of this effect. A search of the CSD with threshold values for the relevant geometric parameters (*e.g.* an external angle <100°) is straightforward but needs to be carried out with care for two reasons. First, significant angular distortions are a necessary consequence of small rings fused to the benzene ring (a four-membered ring has internal angles around 90°), so structures in which *X* is part of a fused ring should be excluded from the search. Second, a surprising number of severely distorted structures (several hundred) are identified, even with few and simple substituents. Closer investigation shows that most of these are structures with probable disorder that has not been handled satisfactorily or some other artefact of a poor refinement model; a particularly common case (almost 30%) is an undoubtedly disordered and poorly modelled toluene solvent molecule, for which a significant distortion is not likely to be real.

Compared with genuine cases of in-plane angular distortions in this way, our own results (Maddock *et al.*, 2018 ▸) are indeed extreme, the smallest C—C—Fe angle in these ferrated benzene derivatives being 97° with others not much larger. Clearly the cause here is a significant secondary Fe⋯N interaction that may be regarded as incipient covalent bonding leading to angular distortions also at the Fe and N atoms. A similar distortion has been found for a P⋯B interaction in a compound with adjacent phosphane and borane substituents (Cowie & Emslie, 2014 ▸). An even more extreme case occurs with an angle of 85° when one of the tri­methyl­silyl groups is removed from a ferrated benzene to give an anionic species (Clegg & Hevia, 2020 ▸; Fig. 3 ▸, bottom). The Fe⋯N secondary bonding interaction here is obviously strengthened and this raises the related question of what distinguishes a secondary interaction from a covalent bond. To find appropriate answers in scientific research we must make sure we ask the right questions!

Another class of compounds that are expected on simple arguments to have planar molecules are porphyrins. Large folding distortions to give bowl and saddle shapes can be produced by a combination of electronic and steric effects of substituents (Smith *et al.*, 2005 ▸, 2018 ▸; Blake *et al.*, 1998 ▸). Reliable characterization of these structures is challenging in the face of high-*Z*′ values and extensive disorder leading to overall low precision, but the observation of consistent bond length patterns permits an assignment of NH versus N in the porphyrins, even though the H atoms cannot be located in difference electron density maps.

## 3. ‘Added value’ from consistencies and trends in a series of structures and comparison with theoretical models   

**Figure 4:**
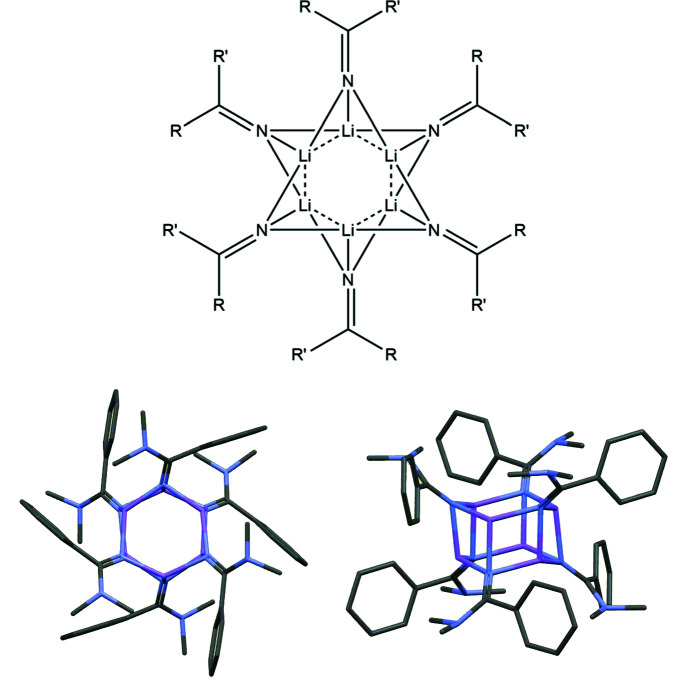
Structural features of hexameric amido­lithium clusters.

Another good example of geometric pattern recognition yielding useful information beyond the statistical significance of a single structure determination is provided by a series of hexameric imidolithium clusters [Li(N=CRR′)]_6_ (Clegg *et al.*, 1983 ▸; Barr *et al.*, 1986 ▸; Armstrong *et al.*, 1987 ▸); though some of these have crystallographic inversion symmetry, others crystallize with more than one molecule in the asymmetric unit, so the entire series gives many instances of a motif of a triply bridging N atom over an Li_3_ triangle (Fig. 4 ▸). The motif is unsymmetrical and in principle has three different, inequivalent Li–N bond lengths; their mean values taken over a total of 24 symmetry-independent units in this series of structures are 1.99, 2.01 and 2.05 Å – differences that are statistically insignificant for individual motifs but are a consistent pattern without exception across all the cases. A theoretical calculation for the archetypal amido­lithium [LiNH_2_]_6_ published at about the same time (Raghavachari *et al.*, 1987 ▸) suggested bond lengths of 1.99, 1.99, and 2.06 Å; the small mismatch with the experimental structures was ascribed by those authors to ‘crystal packing forces’, despite the consistent pattern observed in molecules with different crystalline environments, and led to a discussion in print (Clegg *et al.*, 1988 ▸; Raghavachari *et al.*, 1988 ▸). The theoretical study did not recognize the subtle but important distinction between amido and imido ligand systems; the geometrical distortions away from equal bond lengths are small but significant.

**Figure 5:**
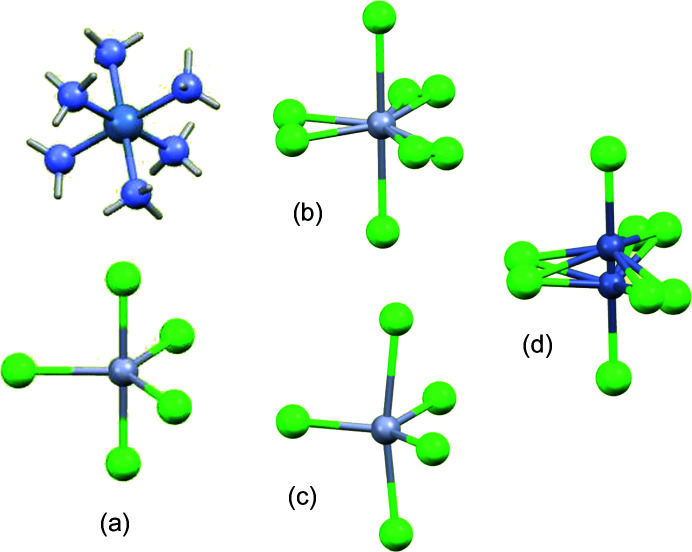
Distortions and disorder of [*M*Cl_5_]^3−^ structures based on a trigonal bipyramid.

An evolving conflict between theoretical and experimental structures was also found in the geometrically simpler case of five-coordinate complex anions [MCl_5_]^3−^, where *M* is a divalent metal, in crystalline salts with an [*M*′(NH_3_)_6_]^3+^ cation (*M*′ = Cr or Co) [Fig. 5 ▸(*a*)]. Previous results with trigonal-bipyramidal geometry were known for *M* = Cu, where shorter axial bonds are an expected consequence of the *d* ^9^ metal ion electron configuration, and for *M* = Cd, where the axial and equatorial bonds are almost the same length; for *d* ^10^ metal ions theoretical models suggested equal bond lengths or an axial elongation (Raymond *et al.*, 1968 ▸; Long *et al.*, 1970 ▸; Burdett, 1975 ▸, 1976 ▸; Rossi & Hoffmann, 1975 ▸). The structure for *M* = Hg, in the same cubic space group as these two, was found to have a marked axial compression in contradiction to this expectation (2.519 versus 2.640 Å) (Clegg *et al.*, 1975 ▸). Subsequent modified theoretical treatments were able to reflect this experimental result (Shustorovich, 1978 ▸). However, a second polymorph with lower symmetry (and minor disorder, easily modelled), discovered later, has the opposite trend, with 3.034 Å axial and 2.417 Å equatorial bonds (Clegg, 1982 ▸), so the situation is not so simple [Fig. 5 ▸(*b*)]. A comparable axial elongation was subsequently found for two different polymorphs of the salt with *M* = Hg and *M*′ = Co (Clegg, 1982 ▸; Herlinger *et al.*, 1981 ▸) [Fig. 5 ▸(*c*)]. The structure of the complex with *M* = Zn and *M*′ = Cr is different again. It is isomorphous with the second (rhombohedral) polymorph of the corresponding Hg complex, but with disorder for the Zn atom as well as the ‘equatorial’ Cl atoms, such that the observed structure represents an intermediate stage of a ligand-exchange reaction between tetrahedral [ZnCl_4_]^2−^ and a further chloride anion (Clegg, 1976 ▸): three (disordered) pseudo-equatorial Zn–Cl bonds are 2.215 or 2.270 Å in length, while the breaking and forming ‘axial bonds’ have lengths of 2.513 and 3.533 Å [Fig. 5 ▸(*d*)].

As well as studying a series of related compounds, valuable information beyond that available from a single-crystal structure can also be derived from measurements on the same sample under different conditions of temperature, pressure or other environmental variables. With modern equipment including diamond anvil cells and highly reliable controlled-temperature devices this is a relatively straightforward undertaking; we used it, for example, in an investigation of the phase transition of barbituric acid dihydrate observed on cooling (Nichol & Clegg, 2005 ▸). It was a much more challenging experiment when Kathleen Lonsdale used variable-temperature data collection with photographic methods (Lonsdale, 1956 ▸), for example, to study atomic and molecular vibrations and thermal expansion for anthra­quinone (Lonsdale *et al.*, 1966 ▸) and for the [2.2′]cyclo­phane molecule di-para-xylylene (Lonsdale *et al.*, 1960 ▸), the latter being another example of benzene ring distortion out of planarity.

## 4. Structural disorder: artefacts, misinterpretation and avoidance   

The incidence of disorder in a crystal structure, as well as complicating the process of structure determination from diffraction data, can lead to problems and ambiguities in interpreting the resulting refinement model. These issues may arise from questions of how the various disorder components should be considered as belonging to the same or different combinations, and also from the possibility that the disorder modelling is inappropriate or incomplete. In some cases, of course, where disorder is likely to be present but has not been recognized, the structure may be seriously misinterpreted. One of the classic examples is the saga of the so-called ‘bond-stretch isomers’ of molybdenum complexes, elegantly summarized by Parkin (1993 ▸). What appeared to be markedly different Mo—O bond lengths in what were otherwise essentially identical molecules were actually artefacts of unrecognized and unresolved disorder of oxo (O) and chloro (Cl) ligands in a solid-solution mixture of two different compounds.

**Figure 6:**
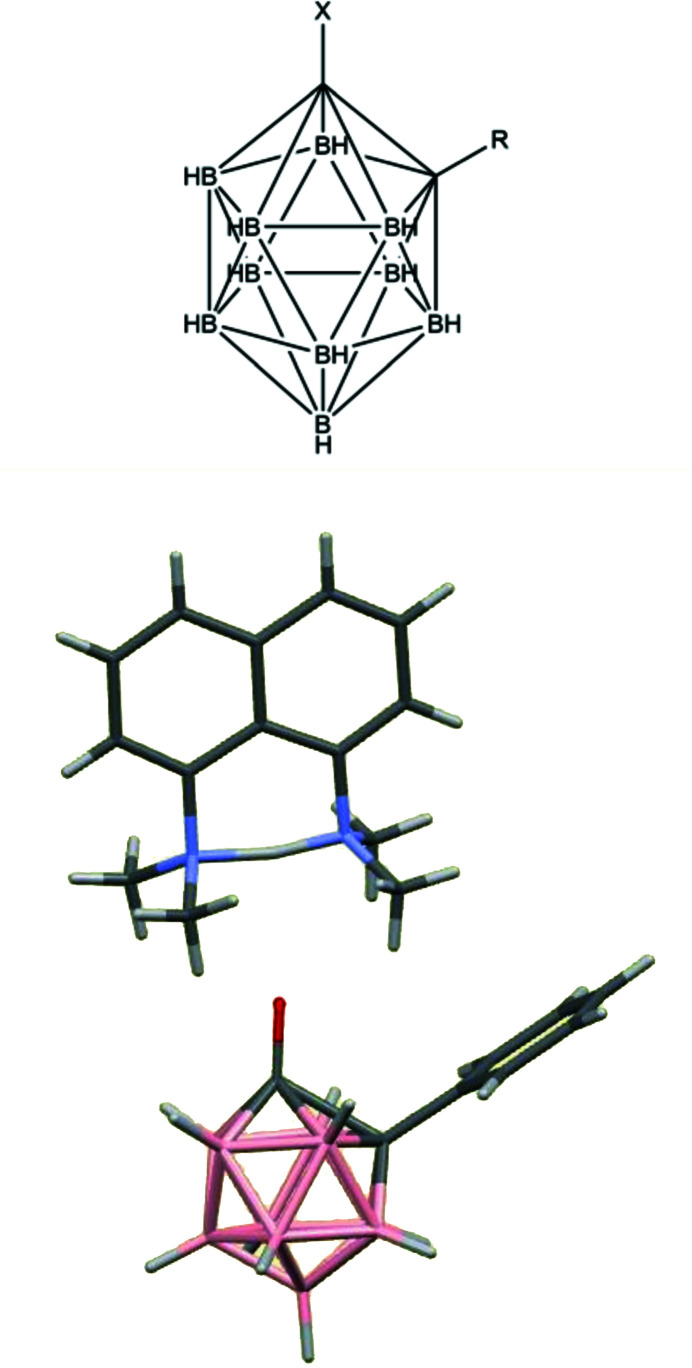
Substituted 1,2-dicarbadodecaboranes.

Disorder can be a particular nuisance for molecules with a degree of pseudo-symmetry and may thwart the whole purpose of a structure determination experiment. An especially good example in my research experience is the investigation of carbaboranes with the intention of finding the structural consequences, particularly the influence on bond lengths, of introducing substituents with different electron-donating characteristics (Fig. 6 ▸). It was expected that the use of a range of substituents (*X*) on one of the two C atoms would have a particularly marked effect on the length of the C—C bond, which is similar to that of the other cage C—B and B—B bonds in many compounds of this family. Unfortunately, initial attempts in which the second C atom remained unsubstituted and retained its terminal H atom led to structures in which this C atom and the four B atoms bonded to the substituted C atom were disordered, there being no crystallographic evidence from geometry or electron densities to distinguish among these five atoms. The disorder, a consequence of five possible orientations of the molecule, is avoided by replacing the carbon-bound H atom by a substituent that is ‘innocent’ in the sense of having no significant electronic influence, thus clearly marking the C atom and, at the same time, providing a steric factor discouraging disorder. The use of a phenyl substituent has the added bonus of improving crystallization by offering the prospect of intermolecular aromatic ring-stacking interactions. The results for a series of compounds with strongly electron-donating substituents (*X*) are unambiguous and very marked, with a considerable elongation of the C—C bond, to as much as 2.001 Å (from around 1.7 Å) for a deprotonated OH substituent that behaves essentially as a pentuply bridging carbonyl group in the cage (Brown *et al.*, 1987 ▸; Coult *et al.*, 1992 ▸; Boyd *et al.*, 2004 ▸; Fox, MacBride *et al.*, 2009 ▸; Fox, Peace *et al.*, 2009 ▸); the ‘proton sponge’ salt of this anion is shown in Fig. 6 ▸.

**Figure 7:**
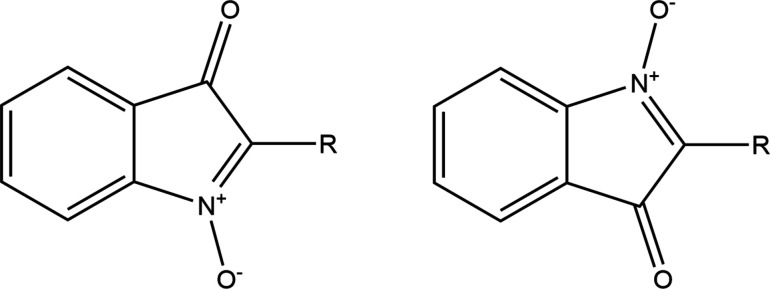
Substituted isatogens with potential disorder of two orientations.

**Figure 8:**
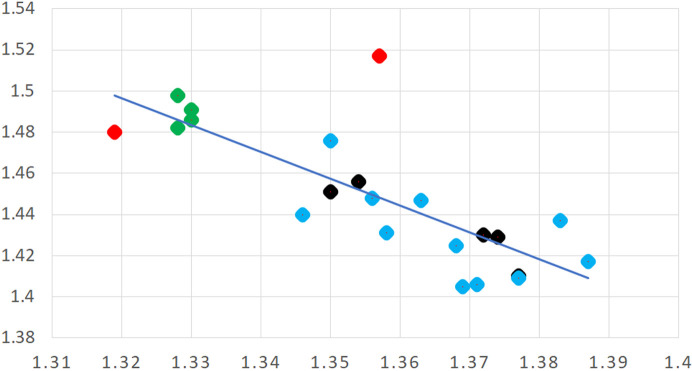
Scatterplot of reported isatogen C—C (vertical axis) and N=C (horizontal axis) bond lengths (Å) for 21 structures in the CSD

A similar approach has met with less success in the case of some isatogens (Fig. 7 ▸), bioactive isomers of isatins. The main structural interest here is the five-membered ring with its two attached O atoms, and the effect of different substituents (*R*) on its electronic and hence geometrical structure. A total of 21 isatogen structures are found in the CSD; 8 of them have been published in journal articles (Adams *et al.*, 1986 ▸, 1990 ▸; Błaszczyk *et al.*, 2006 ▸; Söderberg *et al.*, 2009 ▸; Kirk *et al.*, 2017 ▸), with 13 CSD communications (refcodes: GOGBUB HODXUV HODYAC SAWVAO SAWVES SAWVIW SAWVOC SAWXEU SAWXIY SAWXOE SAWXUK SAWYAR SAZQUI), most of which report disorder. The problem here is that at least one substituent *R*′ is required on the fused benzene ring in order to be completely sure which is the carbonyl group and which the nitroxide in an X-ray crystal structure determination, and that the fused ring system is ordered; otherwise a 180° rotation about the C–*R* bond generates the potential for disorder that is not easily resolved because of the similarity of the electron density of carbon and formally positive nitro­gen atoms and the small differences in expected bond lengths – there are two possible orientations in which the CO and NO groups are exchanged along with the double and single bond connecting them in the five-membered ring. Only 4 of the 21 structures in the CSD have such a substituent *R*′ to ensure structural ordering; for these the difference in the N—O and C=O bond lengths ranges from 0.047 to 0.054 Å, while a larger difference, 0.154–0.170 Å is found for the intervening N=C and C—C bonds. A scatterplot of the C—C versus N=C bond lengths is shown in Fig. 8 ▸. The four *R*′-substituted and thus ordered molecules are represented by green points; they clearly have very similar geometry in this respect and display the largest bond length differences. The two red points are symmetry-independent molecules of one crystal structure that has been solved and refined as non-centrosymmetric but strongly pseudo-centrosymmetric with relatively high *R* factors and imposed restraints (Kirk *et al.*, 2017 ▸); this model must be regarded with some suspicion. All the other structures (black and blue points) have smaller bond length differences that, along with the green points, follow an obvious general trend, which could be interpreted as an electronic effect of substituents. However, the exact same effect would be produced by the type of disorder described above, which leads to a partial averaging of the lengths of these chemically inequivalent bonds; such disorder is explicitly described as partially modelled for 11 of the 16 structures (these 11 are represented by blue points; two points, one blue and one black, are almost completely coincident) and must be regarded as probable for the others, negating any attempt to draw conclusions about the detailed geometry and bonding of the isatogen system and the influence of substituent *R*.

## 5. Crystal structure validation and some selected errors   

Examples were cited of earlier structures found in the CSD which have significant deviations from the expected geometry likely to be artefacts of unresolved disorder or some other defect of the structural refinement model. Although such suspect results might be tolerated from historical studies using what are now obsolete and superseded equipment and methods, there is really no excuse for them in modern X-ray crystallography; nor would they occur if all practitioners of the subject had the thorough approach of crystallographic champions such as Kathleen Lonsdale. The technique inherently has a number of characteristics making it very reliable when appropriately used, to which a range of available tools for checking and validation are added. It has generally always been the case that, given significant diffraction intensity to an appropriate resolution (a generally accepted desirable minimum resolution for chemical crystallography is approximately 0.84 Å, corresponding to measuring diffraction patterns up to a Bragg angle of 25° with Mo *K*α radiation and 67° with Cu *K*α radiation), the number of symmetry-independent reflections in the unique set of data is many times the number of refined parameters in a typical refinement model; the ratio of data to parameters in this so-called overdetermined problem is usually at least 6–8, even if Friedel pairs are averaged for a non-centrosymmetric structure having negligible resonant scattering so that there is no significant difference between the intensities of reflections *hkl* and 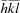, and may be as high as 20 or more with modern equipment. With an appropriate refinement model this high data/parameter ratio leads to low standard uncertainties on the refined parameters, *i.e.* high precision. The measurement of a high ‘multiplicity of observations’ (also known as redundancy, the collection of symmetry-equivalent data and of the same reflections in different geometrical diffractometer settings) also provides a consistency check on the data as well as information that can be used to detect and correct for systematic effects such as absorption.

Structure validation involves checking a refined crystal structure for internal consistency and also comparison with expected results (we have huge accumulated experience of what might be called ‘chemical sense’ in looking at a molecular structure) and with related known structures. The topic of validation has been addressed recently in an educational conference session (Spek, 2020 ▸). Comprehensive and reliable software tools are available for this purpose. These include *PLATON* (Spek, 2003 ▸), which performs internal consistency checks and some comparisons with expected behaviour, raising ‘alerts’ with different levels of severity if potential issues are identified; *CheckCIF*, an online implementation of *PLATON* with additional functionality provided by the IUCr with particular use as a pre-publication check (Spek, 2009 ▸); *Mogul* (Bruno *et al.*, 2004 ▸) for comparison of molecular geometry features with those found in similar structural environments in the CSD to identify unexpected deviations; and specific user-generated searches of the CSD for particular features of interest, which can then be visualized and examined in detail by the graphics and analysis program *Mercury* from the CCDC (Macrae *et al.*, 2006 ▸, 2020 ▸).

Some of these and related validation tools are used for all new entries included in the CSD, with correction of obvious errors, consultation of authors and contributors to deal with others and flags for those that cannot be resolved. Many of the corrections and flagged errors for earlier entries arose from mistakes made manually in transcribing information between computer programs and in publication manuscripts, but these are now rare, particularly since the virtually universal adoption of the CIF standard for archiving and transferring crystal structure results. Other previous potential pitfalls that are now much less likely with integrated software packages and better interfaces between computer programs include the transformation of a unit cell from an initial setting to a different one for reasons of convention or convenience without the corresponding transformation of reflection indices, or with a non-matching transformation. In this context it should be noted that refined fractional atomic coordinates are derived essentially from the reflection intensities, but that the molecular geometry then involves calculations combining these coordinates with the unit-cell parameters, so if these do not match correctly the resulting geometry is distorted, even if the refinement statistics based on measured and calculated intensities are excellent. It is worth remarking that the small number of entries in the CSD from Kathleen Lonsdale’s work, for which atomic coordinates are recorded, generate no significant *Mogul* or *PLATON* alerts beyond those that would be expected for results derived from photographic data collection methods.

Perhaps one of the easiest mistakes to make in a refinement model, particularly when the compound being studied proves to be different from the one expected, is that of wrongly assigned atom types. This means the wrong atomic scattering factor is used for one or more of the atoms in the structure, corresponding to an incorrect electron density. The refinement attempts to compensate for this, mainly by adjusting the displacement parameters, though there may also be an impact on the atomic position and hence the molecular geometry. Such a mistake may be revealed in a number of ways in structure validation. These include unusual bond lengths and/or angles for the atom concerned and its neighbours; unexpectedly large differences in displacement parameters of bonded atoms, including the so-called Hirshfeld ‘rigid bond’ test (Hirshfeld, 1976 ▸); and residual electron density peaks and holes around the misidentified atom. An example from a manuscript submitted for publication and rejected because of these errors (correction of which demonstrated that the structure was already known) was described recently in the *IUCr Newsletter* (Clegg, 2020 ▸): a putative carb­oxy­lic acid was in fact a nitro group, and the chemically highly unlikely tri­hydroxy­methyl substituent should have been tri­fluoro­methyl. The validation alerts for this incorrect structure included impossible hydrogen bonding interactions as well as Hirshfeld test infringements. Another case I encountered as an Editor of *Acta Crystallographica Section E* in the early years of the journal was the claim of an unprecedented one-coordinate copper atom attached to only a single ligand; closer inspection demonstrated that the ‘copper’ atom was almost certainly bromine. A recent thorough analysis of one probable error of this kind, with several misidentified atoms including the rather extreme case of cadmium instead of rhenium (Amemiya *et al.*, 2020 ▸), also gives an extensive list, in its references 66–68, of other known examples.

Other inappropriate structural models and refinement techniques, leading to results that may constitute incorrect structures and raise validation alerts, include unsuitably applied constraints or restraints, particularly in the placement and treatment of hydrogen atoms.

## 6. ‘Alternative facts’: scientific fraud   

Although a misassigned atom type (the wrong element) may be a genuine mistake resulting from ignorance, incompetence or wishful thinking, there have unfortunately been a number of cases where it has been part of deliberate fraud in which falsified results have been submitted for publication, sometimes successfully until the abuse was uncovered by careful validation processes. The first large-scale scandal of this type involved an extensive series of essentially invented crystal structures in which different metal atoms were substituted into the refinement models of previous, genuinely determined structures. In the most blatant cases, exactly the same set of diffraction data was used for the refinement of more than one complex, the differences among electron densities of neighbouring lanthanides, for example, being very small; a little more subtlety was employed in making minor changes to the data and/or the unit-cell parameters at the same time as exchanging the metal. A similar approach was used to ‘substitute’ atoms or chemical groups in organic structures, such as CH_2_ for NH or nitro for carboxyl­ate. This extensive fraud operation, its discovery and consequences, were reported in an editorial by Harrison *et al.* (2010 ▸). It led to a considerable number of retractions of published articles, and corrective action was also required for the corresponding entries in the CSD (Groom, 2010 ▸).

Alerted by this shocking development, the editors of other journals carried out investigations of their own publications, and several fabricated macromolecular structures, some of them of considerable importance and published in internationally leading journals such as *Nature* and *Cell*, were identified and had to be retracted from the Protein Data Bank (PDB) (Dauter & Baker, 2010 ▸); while mistakes may occur in the interpretation of such complex structural problems, in this case it appears that no experimental data actually existed and the false structures were pure inventions.

We are fortunate as crystallographers to have tools available for detecting such nefarious behaviour; X-ray crystallography is, to some extent, a self-checking technique because of the nature and the volume of the diffraction data required for a crystal structure determination. Fraud in many other scientific disciplines must be much harder to detect.

This distasteful and, almost certainly extremely rare, fraudulent behaviour brings us back in conclusion to the qualities of honesty, openness and humility valued and encouraged in scientists by Kathleen Lonsdale, herself a suitable role model for each new generation of crystallographers. These are particularly highlighted in her *Nature* article entitled *Science and Ethics* (Lonsdale, 1962 ▸) – an account that should be read and absorbed by all scientists and also by politicians who currently claim to be ‘following science’ in making their decisions – and would surely be part of the suitable training she proposed should be given to young crystallographers (Lonsdale, 1953 ▸) – a call that is as relevant now as it was almost 70 years ago and is being addressed in part by courses and schools run by the IUCr and its adhering regional and national associations.
